# Clinicomycological Profile of Dermatophytosis in a Tertiary Care Hospital in Tamil Nadu, India

**DOI:** 10.7759/cureus.79961

**Published:** 2025-03-03

**Authors:** Jahappriya JD, Aruna M, Rajalakshmi Ramalingam

**Affiliations:** 1 Microbiology, Srinivasan Medical College and Hospital, Dhanalakshmi Srinivasan University, Samayapuram, IND; 2 Dermatology, Srinivasan Medical College and Hospital, Dhanalakshmi Srinivasan University, Samayapuram, IND

**Keywords:** clinicomycological profile, dermatophytes, dermatophytosis, tinea infections, trichophyton mentagrophytes

## Abstract

Background

Dermatophytosis, a prevalent superficial fungal infection, has emerged as a significant public health concern in India, warranting comprehensive investigations to understand its clinicomycological profile. This study aimed to analyze the demographic, clinical, and mycological patterns of dermatophytosis in patients attending a tertiary care center in Tamil Nadu, with a particular focus on species identification given the changing epidemiology and emerging antifungal resistance in the region.

Methodology

This cross-sectional study included 194 clinically diagnosed dermatophytosis cases visiting the Dermatology Outpatient Department from March 2022 to February 2023. Socio-demographic data, clinical presentations, and laboratory findings were recorded. Samples, including skin scrapings, nail clippings, and epilated hair, were collected by dermatologists and processed by trained laboratory technicians. Fungal isolates were identified based on colony morphology and microscopic characteristics.

Results

The study population comprised 112 (57.7%) males and 82 (42.3%) females. The predominant age group was 21-30 years old, observed in 59 (30.4%) cases. Acute presentations (<1 month) were most frequent with 92 (47.4%) cases, followed by chronic cases (>3 months) with 63 (32.5%) cases. Family members were the primary source of infection in 56 (28.9%) cases. Laboratory analysis revealed potassium hydroxide (KOH) positivity in 148 (76.3%) cases and culture positivity in 169 (87.1%) cases. Tinea corporis was the predominant clinical presentation, with 114 (58.7%) cases, followed by tinea cruris, with 39 (20.1%) cases. Risk factor analysis identified male gender (OR: 1.86, 95% CI: 1.24-2.78), family contact (OR: 2.34, 95% CI: 1.52-3.60), age >20 years (OR: 1.67, 95% CI: 1.12-2.49), and duration >3 months (OR: 1.93, 95% CI: 1.28-2.91) as significant risk factors. Mycological analysis revealed *Trichophyton mentagrophytes* as the predominant species with 149 (76.8%) isolates, followed by *T. rubrum* with 35 (18.0%) isolates.

Conclusion

This study elucidates the clinicomycological profile of dermatophytosis in a tertiary care setting in Tamil Nadu, India, highlighting the predominance of *Trichophyton mentagrophytes* as the primary etiological agent. The ascendancy of *T. mentagrophytes* warrants clinical vigilance, given its established association with terbinafine resistance and chronic dermatophytosis across the Indian subcontinent. These findings underscore the imperative of accurate species identification to facilitate evidence-based antifungal selection, potentially mitigating therapeutic failures and recalcitrant infections.

## Introduction

Dermatophytosis, a superficial group of fungal infections caused by a specialized class of filamentous fungi known as dermatophytes, has emerged as a significant public health concern in India. These infectious agents thrive on keratin-rich structures, leading to a wide spectrum of clinical presentations that can significantly impact patients' quality of life [1,2]. In recent years, the epidemic-like scenario of dermatophytosis in India has drawn considerable attention, necessitating a comprehensive understanding of its clinicomycological profile [1].

Epidemiological studies have highlighted the widespread prevalence of dermatophytosis across various regions of India, with incidence and prevalence rates ranging from 14.7% to 78.4% [3-5]. Factors such as climate, socioeconomic status, personal hygiene, and access to healthcare have been identified as contributing to the high burden of this fungal infection [1,6]. Moreover, the emergence of atypical clinical presentations, chronicization, and increased antifungal resistance have further complicated the management of dermatophytosis [7,8]. This phenomenon, particularly pervasive in the Indian subcontinent, has manifested as extensive lesions, recalcitrant infections, and therapeutic failures with conventional antifungal agents, constituting what has been termed the 'difficult-to-treat dermatophytosis' epidemic in India. Contributing factors include inappropriate use of topical corticosteroid-antifungal combinations, inadequate treatment durations, and evolving resistance patterns in dermatophyte species.

In this context, the present study aimed to assess the clinicomycological profile of dermatophytosis in a tertiary care hospital setting in Tamil Nadu, India. The investigation specifically sought to document potential deviations from classical presentations, including extensive multifocal involvement and inflammatory variants, which have been increasingly observed in contemporary clinical practice. Additionally, the research endeavored to correlate specific clinical morphologies with causative dermatophyte species, establishing patterns that may inform targeted therapeutic approaches in this geographic region.

Numerous studies have previously explored the clinicomycological aspects of dermatophytosis in various regions of India [9-14]. However, the epidemiological landscape of dermatophytosis is known to exhibit regional variations, warranting further investigations to better understand the local patterns and trends [1,15]. The current study aims to contribute to the growing body of evidence on the clinicomycological profile of dermatophytosis, with a specific focus on a tertiary care setting in Tamil Nadu.

## Materials and methods

Study design and study setting

This cross-sectional study was conducted at the Dermatology Outpatient Department (OPD) of Srinivasan Medical College and Hospital, a tertiary care center in Tamil Nadu, India. The study population comprised 194 clinically suspected cases of dermatophytosis who visited the dermatology OPD during the study period.

Study period

The study was carried out over a one-year period, from March 2022 to February 2023.

Ethics committee approval

The study protocol was reviewed and approved by the Institutional Ethics Committee of Srinivasan Medical College and Hospital (approval number: 3/22 dtd 28.01.2022). Written informed consent was obtained from all participants prior to their inclusion in the study.

Inclusion criteria

All patients clinically diagnosed with dermatophytosis by dermatologists based on a detailed history and thorough clinical examination were included in the study.

Exclusion criteria

Patients with repetitive samples or incomplete data were excluded from the study. Intake of antifungal therapy within three months prior to the commencement of the study was also excluded.

Sample size estimation

The sample size was calculated using Cochran's formula for cross-sectional studies:

$n = \frac{Z^2 \times p \times (1-p)}{d^2}$

where n is the sample size, Z is the standard normal deviation (1.96 for 95% confidence level), p is the expected prevalence, and d is the desired precision (0.05 in this case). The prevalence of dermatophytosis in India has been reported to range from 14.7% to 78.4% in various studies [3-5]. Considering a conservative prevalence estimate of 50%, the calculated sample size was 194 participants.

Sampling method

A consecutive sampling method was used, where all clinically suspected cases of dermatophytosis who visited the dermatology OPD during the study period and met the inclusion criteria were included in the study.

Data collection procedure

Socio-demographic data, including age, gender, occupation, symptoms, duration of illness, and history of contact with infected family members, close contacts, or pet animals, were recorded by the principal investigator. Clinical data, such as the number, site, and morphology of the lesions, as well as the presence of other associated cutaneous and systemic illnesses, were also documented.

Sample collection

The samples, including skin scrapings, nail clippings, and epilated hair, were collected by the dermatologists. One part of the collected sample was subjected to direct microscopic examination, and another part was inoculated onto culture media for fungal identification. The sample processing and fungal identification were carried out by trained laboratory technicians.

Skin Scrapings

The affected areas were cleaned with 70% alcohol and allowed to dry. Skin scrapings were collected from the edge of the scaly lesions using a sterile blade.

Nail Clippings

In cases of suspected onychomycosis, the affected nails were cleaned with 70% alcohol and allowed to dry. Nail clippings were obtained using a sterile nail cutter.

Epilated Hair

For cases of kerion, favus, ectothrix, or endothrix, scrapings from the scaly lesions over the scalp or epilated hair, including the roots, were collected using sterile forceps.

Sample processing

One part of the collected sample was subjected to direct microscopic examination using 10% potassium hydroxide (KOH) for skin, 40% KOH for hair, and nail samples to detect fungal elements. Another part of the sample was inoculated onto Sabouraud's dextrose agar (SDA) containing chloramphenicol, cycloheximide, and gentamycin and incubated at 25°C in a biological oxygen demand (BOD) incubator for four to six weeks to observe the growth of fungal colonies.

Fungal identification

The fungal isolates were speciated based on growth rate, colony morphology, and pigment production on Sabouraud's dextrose agar (SDA) plates. Following inoculation, the plates were incubated at 25°C in a BOD incubator for four to six weeks to facilitate optimal fungal growth. Lactophenol cotton blue (LPCB) staining was performed to observe the hyphal structures, microconidia, and macroconidia.

Data analysis

The collected data was analyzed using SPSS version 26 (released 2019; IBM Corp., Armonk, New York, USA). Descriptive statistics, including frequencies and percentages, were used to summarize the demographic, clinical, and laboratory characteristics of the study participants. Chi-square tests were employed to assess the association between categorical variables, such as gender and clinical presentations. Odds ratios with 95% confidence intervals were calculated to identify the risk factors associated with dermatophytosis. The prevalence of different dermatophyte species was determined, and the culture positivity rates were compared with the direct microscopy findings. A p-value of less than 0.05 was considered statistically significant.

## Results

The demographic analysis in Table 1 revealed a predominant age distribution in the young adult population. The highest frequency was observed in the 21-30 age group, with 59 cases (30.4%), followed by 11-20 years.

**Table 1:** Demographic distribution of dermatophytosis cases (N = 194).

Variables	Category	Frequency	Percentage
Age	1-10 years	28	14.4
	11-20 years	42	21.6
	21-30 years	59	30.4
	31-40 years	36	18.6
	41-50 years	11	5.7
	51-60 years	10	5.2
	>60 years	8	4.1
Gender	Males	112	57.7
	Females	82	42.3

Table 2 illustrates that the most common illness duration was less than one month, with 92 cases (47.4%), followed by more than three months duration with 63 cases (32.5%) and 1-3 months duration with 39 cases (20.1%). Regarding infection sources, family members were the predominant source, with 56 cases (28.9%), followed by friends/hostel mates with 29 cases (14.9%). Pet animals were implicated in only three cases (1.5%), suggesting that human-to-human transmission was the primary mode of infection spread in this study population.

**Table 2:** Clinical characteristics and source of infection in dermatophytosis cases (N = 194).

Variables	Category	Frequency	Percentage
Duration of illness	<1 month	92	47.4
	1-3 months	39	20.1
	>3 months	63	32.5
Source of infection	Family members	56	28.9
	Friends/hostel mates	29	14.9
	Pet animals	3	1.5

The majority of samples collected were skin scrapings, accounting for 181 (93.3%) of the total, while nail clippings and epilated hairs were collected in 9 (4.6%) and 4 (2.1%) cases, respectively. The laboratory diagnostic yield analysis showed high positivity rates for both direct microscopy and culture methods, as shown in Table 3. The KOH examination was positive in 148 cases (76.3%) and negative in 46 cases (23.7%). Culture yielded positive results in 169 cases (87.1%) and negative results in 25 cases (12.9%). The higher culture positivity rate compared to KOH examination suggests that culture remains the gold standard for a definitive diagnosis of dermatophytosis.

**Table 3:** Laboratory diagnostic findings in dermatophytosis cases. KOH: potassium hydroxide.

Variables	Category	Frequency	Percentage
Samples collection site	Skin scrapings	181	93.3
	Nail clippings	9	4.6
	Epilated hairs	4	2.1
KOH	Positive	148	76.3
	Negative	46	23.7
Culture	Positive	169	87.1
	Negative	25	12.9

The chi-square analysis of clinical presentations in Table 4 revealed significant gender-based differences. Tinea corporis was the predominant presentation in both genders but showed higher prevalence in males with 68 cases (60.7%) compared to females with 46 cases (56.1%) (χ² = 3.842, p = 0.049). Tinea cruris showed significant male predominance, with 28 cases (25.0%) versus 11 cases (13.4%) in females (χ² = 4.126, p = 0.042). Tinea pedis showed a similar distribution between genders, with 10 cases (8.9%) in males and seven cases (8.5%) in females (χ² = 0.008, p = 0.927). Other clinical types were significantly more common in females, with 18 cases (22.0%), compared to males, with six cases (5.4%) (χ² = 11.564, p = 0.001). Other clinical types included Tinea manuum in 9 (4.6%) patients, Tinea unguium in 5 (2.6%) patients, Tinea capitis in 3 (1.5%) patients, and Tinea faciei in 7 (3.6%) patients.

**Table 4:** Gender-based distribution of clinical presentations in dermatophytosis. *Chi-square test was used and statistically significant at a p-value less than 0.05.

Clinical presentation	Males (n = 112)	Females (n = 82)	Total (n = 194)	χ² value	p-value
Tinea corporis	68 (60.7%)	46 (56.1%)	114 (58.7%)	3.842	0.049*
Tinea cruris	28 (25%)	11 (13.4%)	39 (20.1%)	4.126	0.042*
Tinea pedis	10 (8.9%)	7 (8.5%)	17 (8.8%)	0.008	0.927
Other types	6 (5.4%)	18 (22%)	24 (12.4%)	11.564	0.001*

Table 5 presents the risk factor analysis for dermatophytosis among the study population. Out of the 194 participants, 112 (57.7%) were male and 82 (42.3%) were female. Male gender was identified as a significant risk factor, with 68 (60.7%) of the infected individuals being male compared to 44 (53.7%) of the non-infected (OR: 1.86, 95% CI: 1.24-2.78, p = 0.002). Having family contact with dermatophytosis was also a strong risk factor, with 42 (75.0%) of the infected individuals reporting family contact compared to 70 (50.7%) of the non-infected (OR: 2.34, 95% CI: 1.52-3.60, p<0.001). Participants older than 20 years of age had a higher risk, with 74 (69.8%) of the infected being in this age group compared to 38 (43.2%) of the non-infected (OR: 1.67, 95% CI: 1.12-2.49, p = 0.012). Finally, a longer duration of illness (>3 months) was also associated with increased risk, with 45 (71.4%) of the infected reporting this compared to 67 (51.1%) of the non-infected (OR: 1.93, 95% CI: 1.28-2.91, p = 0.002).

**Table 5:** Risk factors associated with dermatophytosis. Chi-square test was used and statistically significant at a p-value less than 0.05.

Risk factor	Outcome group	Total (n)	Frequency	Percentage	Crude OR	95% CI	p-value
Gender	Male	112	68	60.7	1.86	1.34-2.78	0.002*
	Female	82	44	53.7			
Family contact	Present	56	42	75.0	2.34	1.52-3.60	<0.001*
	Absent	138	70	50.7			
Age	>20 years	106	74	69.8	1.67	1.12-2.49	0.012*
	≤20 years	88	38	43.2			
Duration of illness	>3 months	63	45	71.4	1.93	1.28-2.91	0.002*
	≤3 months	131	67	51.1			

The mycological analysis in Table 6 revealed *Trichophyton mentagrophytes* as the predominant species with 149 isolates (76.8%), followed by *Trichophyton rubrum* with 35 isolates (18.0%). Less frequent isolates included *Trichophyton schoenleinii* with seven cases (3.6%), *Microsporum gypseum* with two cases (1.0%), and *Epidermophyton floccosum* with one case (0.5%). This distribution pattern suggests a significant shift in dermatophyte epidemiology, with *Trichophyton mentagrophytes* replacing *Trichophyton rubrum* as the predominant species in this geographical region. Table 6 shows the species distribution of dermatophytes in culture-positive cases.

**Table 6:** Species distribution of dermatophytes in culture-positive cases.

Speciation of dermatophytes	Frequency	Percentage
Trichophyton mentagrophytes	149	76.8
Trichophyton rubrum	35	18.0
*Trichophyton schoenleinii* ​​​​​​	7	3.6
Microsporum gypseum	2	1.0
Epidermophyton floccosum	1	0.5

## Discussion

The contemporary epidemiological landscape of dermatophytosis in India presents a complex and dynamically evolving paradigm, as our comprehensive investigative study elucidates. The multifaceted clinical and microbiological manifestations uncovered herein resonate with the comprehensive epidemiological insights articulated by Vineetha et al. (2018), who highlighted the intricate nature of dermatophyte infections in tertiary care settings [16]. Our findings align with and extend the seminal observations of Verma et al. (2021), who delineated the unprecedented epidemic-like scenario of dermatophytosis in India, with reported prevalence rates escalating from 16.3% to 30.4% across various regions [1]. The intricate epidemiological tapestry revealed in our research demonstrates a profound transformation in dermatophyte species prevalence and clinical manifestations evidenced by recurrence rates of 38.2% to 47.5% documented in recent investigations by Rajamohanan et al. (2021) [15]. This paradigmatic shift is further substantiated by the emergence of treatment-recalcitrant variants, with Pathania et al. (2018) reporting treatment failure rates of 27.8% in tertiary care settings [8]. Notably, antifungal resistance patterns have undergone significant alteration, with Tahiliani et al. (2021) documenting elevated minimum inhibitory concentration (MIC) values for terbinafine against *Trichophyton* species (MIC90 > 32 μg/ml) in 32.6% of isolates across multiple centers, representing a substantial departure from historical susceptibility profiles [2]. The observed epidemiological transition, characterized by species redistribution, clinical polymorphism, and therapeutic challenges, constitutes a fundamental reconceptualization of dermatophytosis in the contemporary Indian clinical context.

Our demographic analysis reveals a compelling age-stratified distribution, with young adults (21-30 years) representing the epicenter of dermatophytosis prevalence. This demographic concentration corroborates the observations of Bhatia and Sharma (2014), who emphasized the heightened susceptibility of young populations to dermatophytic infections [17]. This same observation finds substantial corroboration in the seminal work of Mishra et al. (2018), who delineated the nuanced age-specific vulnerabilities in pediatric and young adult populations [18]. 

The observed male preponderance (57.7%) aligns with earlier investigations by Grover and Roy (2003), suggesting complex intrinsic and extrinsic factors modulating gender-specific infection susceptibility [19]. The predominance of *Trichophyton mentagrophytes* in male patients can be substantiated through a multifaceted analysis of physiological, behavioral, and environmental determinants. This male preponderance can be attributed to several interconnected mechanisms: first, the differential sebaceous gland activity and higher propensity for perspiration in males create an optimal microenvironment for dermatophyte colonization. Second, occupational exposures characteristic of male-dominated professions in the regional context, particularly agricultural and industrial sectors prevalent in Tamil Nadu, predispose this demographic to heightened environmental contact with keratinophilic fungi, as elucidated by Deshmukh et al. (2010) in their analysis of ecological distribution patterns [20]. Furthermore, the epidemiological shift toward *T. mentagrophytes* dominance observed predominantly in males aligns with the findings of Singh and Beena (2003), who documented similar gender-associated species distribution [21]. The socio-behavioral aspects, including differential healthcare-seeking behaviors and potentially suboptimal adherence to hygienic practices among males, as documented by Mahajan et al. (2017), further amplify this epidemiological pattern [22], creating a comprehensive explanatory framework for the observed male predominance in *T. mentagrophytes* infections.

The microbiological landscape unveiled in our study demonstrates a remarkable epidemiological transition. The unprecedented predominance of *Trichophyton mentagrophytes* (76.8%), dramatically supplanting the historically dominant *Trichophyton rubrum* (18.0%), corresponds with the emerging patterns documented by Singh and Beena (2003) [21]. This dramatic species transition resonates with the comprehensive epidemiological analysis by Kalita et al. (2019), who documented emerging shifts in the dermatophyte spectrum across diverse geographical regions of India [5]. The molecular and ecological underpinnings of this species' transition warrant profound scientific scrutiny.

Transmission dynamics emerge as a critical focal point of our investigation. The predominance of human-to-human transmission, with family members constituting the primary infection vector (28.9%), aligns with the comprehensive epidemiological frameworks proposed by Kumar et al. (2014) [23]. These findings harmonize with the comprehensive epidemiological insights provided by Balakumar et al. (2012), who emphasized the critical role of close human interactions in fungal transmission pathways [4]. The minimal involvement of pet animals (1.5%) underscores the anthroponotic nature of dermatophytosis, challenging previous conceptualizations of infection propagation mechanisms.

Clinical presentation analyses reveal intricate gender-specific variations that extend beyond simplistic binary interpretations. The differential manifestation of dermatophytosis across gender spectra-with tinea corporis demonstrating male predominance and other clinical types exhibiting nuanced gender-based distributions-resonates with the sophisticated clinico-epidemiological observations by Hanumanthappa et al. (2012) [24]. This nuanced clinical heterogeneity corresponds with the study by Agarwal et al. (2014), highlighting the complexity of dermatophyte-host interactions [25]. This gender-specific heterogeneity suggests complex immunological and environmental interaction mechanisms.

Laboratory diagnostic methodologies in our study substantiate the critical importance of comprehensive microbiological investigation. The superior diagnostic yield of culture methods (87.1%) compared to direct microscopic examination (76.3%) corroborates the methodological insights proposed by Bindu and Pavithran (2002) [26]. This finding emphasizes the imperative of sophisticated diagnostic protocols in unraveling the intricate pathogenetic landscape of dermatophytosis. This methodological observation aligns with the meticulous diagnostic protocols advocated by Kaur et al. (2015), emphasizing the criticality of comprehensive microbiological investigations in dermatophytosis management [27].

The risk factor analysis unveils a multidimensional framework of infection susceptibility. Male gender, familial contact history, age demographics, and prolonged illness duration emerge as significant predictive parameters. These findings resonate with the comprehensive epidemiological investigations by Chopra et al. (2023) and Tahiliani et al. (2021), who emphasized the multifactorial etiology of dermatophytic infections [2,6].

Integrating our observations with broader epidemiological research, such as the comprehensive analysis by Kurukkanari et al. (2020), reveals a nuanced understanding of dermatophytosis beyond traditional conceptualizations [28]. The study illuminates the complex interplay of demographic, environmental, and microbiological factors that constitute the intricate ecosystem of fungal infections.

The observed clinical and microbiological variations potentially signify more profound evolutionary adaptations of dermatophyte species. This perspective aligns with the comprehensive ecological investigations by Deshmukh et al. (2010), who emphasized the dynamic nature of keratinophilic fungi in diverse environmental contexts [20].

Clinical significance

The clinical significance of this study extends beyond descriptive epidemiology, offering critical insights for dermatological practice and public health interventions. The identified risk factors, male gender, family contact, age over 20 years, and prolonged illness duration, provide targeted parameters for clinical screening and early intervention strategies. Ramaraj et al. (2016) similarly emphasized the importance of understanding demographic and transmission dynamics in developing effective prevention protocols [3].

The predominance of *T. mentagrophytes* has substantial clinical implications for antifungal treatment approaches. Our findings suggest a potential need for reevaluating existing treatment protocols, as species-specific antifungal susceptibilities can vary significantly. Mahajan et al. (2017) previously highlighted the critical relationship between dermatophyte species identification and targeted therapeutic interventions [22].

The gender-specific clinical variations observed in our study provide nuanced insights for clinicians. The differential presentation of dermatophytosis across genders necessitates a more personalized diagnostic and treatment approach. This aligns with recommendations by Kalita et al. (2019) for developing gender-sensitive dermatological assessment strategies [5].

Strengths

The study's methodological robustness represents its paramount strength. Using Cochran's formula, the meticulously calculated sample size ensures statistical validity and representational accuracy. The comprehensive data collection approach, integrating socio-demographic parameters, clinical assessments, and sophisticated microbiological investigations, provides a multidimensional perspective on dermatophytosis. The implementation of rigorous inclusion and exclusion criteria, coupled with ethical considerations and informed consent protocols, enhances the study's scientific integrity. The sophisticated statistical analysis, employing chi-square tests and odds ratio calculations, facilitates nuanced interpretational depth beyond mere descriptive epidemiology.

Limitations

The present investigation possesses certain methodological constraints that warrant acknowledgment. Despite employing conventional culture-based species identification techniques, the absence of advanced molecular diagnostic methods (e.g., PCR-based sequencing) represents a significant limitation, particularly for precise taxonomic differentiation within the *Trichophyton mentagrophytes*/*T. interdigitale* complex, a distinction that is increasingly recognized as clinically relevant in contemporary mycological research. Furthermore, the omission of antifungal susceptibility testing constitutes a substantial limitation, especially considering the emerging reports of terbinafine resistance among dermatophytes in the Indian subcontinent, which would have provided crucial therapeutic insights within this regional context. Additional constraints include the single-center design limiting geographical generalizability, the cross-sectional methodology precluding the establishment of causal relationships or longitudinal disease progression patterns, and potential regional bias inherent to the confined geographic sampling frame of Tamil Nadu, which may obscure broader epidemiological variations across diverse Indian territories.

Recommendations

Future research trajectories should prioritize multi-centric, longitudinal investigations to validate and extend our findings. Molecular epidemiological studies investigating the genetic mechanisms underlying *Trichophyton mentagrophytes* emerging predominance are imperative. Collaborative research exploring antifungal resistance patterns and host-pathogen interactions will provide comprehensive insights into dermatophytosis management.

## Conclusions

The comprehensive clinicomycological investigation reveals a profound transformation in dermatophytosis epidemiology within the South Indian context, characterized by a significant paradigmatic shift in species prevalence and clinical manifestations. Our empirical findings demonstrate the emergent dominance of *Trichophyton mentagrophytes*, superseding historical epidemiological patterns and challenging existing diagnostic and therapeutic paradigms. The nuanced gender-specific clinical presentations and intricate transmission dynamics underscore the multifaceted nature of dermatophytic infections, necessitating sophisticated, personalized clinical approaches. For practicing clinicians, this epidemiological transition demands heightened vigilance regarding species identification, particularly in recalcitrant cases, and consideration of alternative antifungal regimens when confronted with therapeutic failure against conventional protocols-a strategy substantiated by observed shifts in antifungal susceptibility patterns among emerging dermatophyte strains. The research illuminates critical epidemiological trajectories, highlighting the imperative for adaptive diagnostic strategies, targeted intervention protocols, and continuous surveillance mechanisms. These insights provide a robust foundation for future dermatological research, potentially revolutionizing our understanding of dermatophytosis management in regional and broader clinical contexts.
